# Should uterus transplants be publicly funded?

**DOI:** 10.1136/medethics-2015-102999

**Published:** 2015-12-15

**Authors:** Stephen Wilkinson, Nicola Jane Williams

**Affiliations:** Department of Politics, Philosophy and Religion, Lancaster University, Lancaster, UK

**Keywords:** Transplantation, Reproductive Medicine, Concept of Health, Allocation of Health Care Resources, Distributive Justice

## Abstract

Since 2000, 11 human uterine transplantation procedures (UTx) have been performed across Europe and Asia. Five of these have, to date, resulted in pregnancy and four live births have now been recorded. The most significant obstacles to the availability of UTx are presently scientific and technical, relating to the safety and efficacy of the procedure itself. However, if and when such obstacles are overcome, the most likely barriers to its availability will be social and financial in nature, relating in particular to the ability and willingness of patients, insurers or the state to pay. Thus, publicly funded healthcare systems such as the UK's National Health Service (NHS) will eventually have to decide whether UTx should be funded. With this in mind, we seek to provide an answer to the question of whether there exist any compelling reasons for the state not to fund UTx. The paper proceeds as follows. It assumes, at least for the sake of argument, that UTx will become sufficiently safe and cost-effective to be a candidate for funding and then asks, given that, what objections to funding there might be. Three main arguments are considered and ultimately rejected as providing insufficient reason to withhold funding for UTx. The first two are broad in their scope and offer an opportunity to reflect on wider issues about funding for infertility treatment in general. The third is narrower in scope and could, in certain forms, apply to UTx but not other assisted reproductive technologies (ARTs). The first argument suggests that UTx should not be publicly funded because doing so would be inconsistent with governments’ obligations to prevent climate change and environmental pollution. The second claims that UTx does not treat a disorder and is not medically necessary. Finally, the third asserts that funding for UTx should be denied because of the availability of alternatives such as adoption and surrogacy.

## Introduction

Since 2000, 11 human uterine transplantation procedures (UTx) have been performed across Europe and Asia.^1–3^ Five of these have, to date, resulted in pregnancy and four live births have now been recorded.^4^ ^5^ UTx is a potential treatment option for the 1 in every 500 women worldwide of childbearing age^6^ with absolute uterine factor infertility (AUFI): an umbrella term covering fertility problems occurring as a result of either a complete lack of a uterus due to congenital abnormality or previous hysterectomy, or the possession of a malformed or diseased uterus.^7^ Individuals with AUFI presently have very limited options for parenthood: essentially adoption or surrogacy.

The most significant obstacles to the availability of UTx are presently scientific and technical, relating to the safety and efficacy of the procedure itself. However, if and when such obstacles are overcome, the most likely barriers to its availability will be social and financial in nature, relating in particular to the ability and willingness of patients, insurers, or the state, to pay. Thus, publicly funded healthcare systems such as the UK's National Health Service (NHS) will eventually have to decide whether UTx should be funded. Given however that even funding for IVF generates public hostility, and is only patchily supported, it is likely that any proposal to fund UTx will be controversial. With this in mind—assuming that UTx becomes a safe and effective treatment for AUFI and that, as time passes, its cost is reduced such that it meets the normal cost-effectiveness threshold laid down by institutions such as the UK's National Institute for Health and Care Excellence (NICE) of £20–30 000 per quality-adjusted life year (QALY)^8^—this paper asks whether there would, in such circumstances, exist any compelling reason for the NHS not to fund UTx.^i^

The paper proceeds as follows. It assumes, at least for the sake of argument, that UTx will become sufficiently safe and cost-effective to be a candidate for funding and then asks, given that, what objections to funding there might be. Three main arguments are considered. The first suggests that UTx should not be publicly funded because doing so is inconsistent with governments’ obligations to prevent climate change and environmental pollution. The second claims that UTx does not treat a disorder and is not medically necessary. Finally, the third asserts that funding for UTx should be denied because of the availability of alternatives such as adoption and surrogacy. Of these, the first two arguments apply to assisted reproductive technologies (ARTs) in general and offer an opportunity to reflect on wider issues about funding for infertility treatment. The third, however, is narrower in scope and could, in certain forms, apply to UTx but not to other ARTs.

## The environmental argument

For each child made through medical intervention a carbon legacy results. ARTs should be allocated with due concern for the environment and sober consideration for the implications of climate change.^9^

Let's allow (at least for the sake of argument) that a legitimate aim of state policy is reducing environmental pollution, such as greenhouse gas emissions, and that reducing or containing population size is a necessary means of achieving this. Would that give the state a reason not to fund UTx? If the answer is ‘yes’ then such considerations tell against funding for ARTs of any kind. UTx is, after all, not uniquely vulnerable to these objections; they apply equally to all IVF, and indeed more pressingly to IVF given the numbers involved.^ii^ This argument is, however, liable to a serious objection.

This is that the costs of preventing climate change and pollution should not be borne disproportionately by the infertile; it would instead be fairer, and perhaps also more effective, to bring about population control in other ways. There could, for example, be ‘tax breaks’ for non-reproducers, or public education programmes aimed at discouraging reproduction. To illustrate the attractiveness of this view, consider these examples.^iii^

***CAS***

Many people suffer from a pathological condition called Commercial Airline Syndrome (CAS), which is caused by a defect in the inner ear. The main symptom of CAS is severe pain during take-off and landing and for several hours afterwards. CAS sufferers find air travel unbearable. CAS can be cost-effectively cured using surgery. However, the government refuses to provide funding on the grounds that doing so would enable people to contribute to carbon emissions and climate change.

***Motability***

The Government financially supports a scheme called Motability, which allows persons with disabilities and/or their families and carers to lease a new car, in some cases one with special adaptations. Opposition politicians however object to this scheme as follows: if an important government objective is to discourage private car use, why are we spending public money to support car use amongst people with disabilities, thereby boosting carbon emissions and adding to congestion?

These examples are meant to generate the intuition that what is proposed is unfair. What seems particularly unfair is that persons with disorders or disabilities have services withheld from them on environmental grounds while others are allowed to continue polluting without suffering anything like the same level of personal cost.

Why is this unfair? One reason is that if discouraging environmental pollution were as important as suggested, then *everyone*, not just those with medical/mobility needs, should be given an incentive not to pollute. So, ‘singling out’ people with disabilities raises the question of whether the environmental reason offered is little more than a thinly veiled attempt to hide the real motive behind such denials of treatment, disability discrimination. A second reason for regarding these cases as unfair is the scale of the costs incurred by those who have medical/mobility assistance withheld. The consequences for such individuals are potentially very serious indeed: not being able to fly or—worse—drive. However, if (extra) ‘carbon taxes’ were levied at a low level on the entire population instead, we could presumably achieve a similar (or greater) reduction in emissions, increase the tax take and spread the pain much more equally, thinly *and* fairly.

These arguments are compelling and apply to the examples above and to the major concerns of this paper: funding for ARTs in general and UTx in particular. Thus, in the case of IVF, if it were argued that the state should not provide funding because of its interest in preventing climate change, we should reply that—if that is its concern—then it should (for example) tax *all* human reproduction rather than singling out those who are pathologically infertile for especially negative treatment. Much the same goes for UTx. We broadly agree therefore with the view put forward by Karnein and Iser that:

The burden of reducing overpopulation has to be distributed equally and cannot be shouldered by those in need of ARTs and reproductive donation alone.^11^

## Is infertility a disease?

A second line of attack on public funding for UTx lies in the suggestion that infertility is not a ‘real’ or ‘proper’ disease. This is an interesting suggestion, although one that immediately runs up against the fact that infertility does enjoy ‘official disease status’. For example, the International Committee Monitoring Assisted Reproductive Technologies (ICMART) and WHO *Revised Glossary of ART Terminology* give the following ‘clinical definition’ of infertility:

A disease of the reproductive system defined by the failure to achieve a clinical pregnancy after 12 months or more of regular unprotected sexual intercourse^12^ ^iv^

However, official definitions notwithstanding, there remains suspicion about the proposal that infertility (*a fortiori* AUFI) *should* be classified as such. That such is the case is demonstrated by the results of a large-scale survey conducted by Adashi *et al* in six European countries, the USA and Australia regarding public perceptions of infertility and its treatment. In response to the statement ‘infertility is a disease’ only 38% of respondents agreed.^14^ As Ravitsky *et al* note regarding this study:

The implications of the question are clear: if perceived as a disease, public funding for its treatment is construed as justified and what remains to be determined is its prioritization in relation to other required treatments competing for limited resources…if not, funding it may not be justified from the outset.^15^

The following comments by Pemberton encapsulate this sort of scepticism:

In the 1960s, those unable to conceive were referred to as the ‘involuntary childless’. Today, this has been reframed within the discourse of biomedicine as ‘infertility’, and it reflects an increasing tendency for medicine to step in to manage and provide solutions to social problems. This, of course, does not detract from the upset that childlessness can bring. But, this is grief based on a sense of failure because of an ‘abnormality’ that is culturally determined. I am not arguing that the infertile should not be free to seek assistance with conception if they choose it. My issue is whether they are entitled to treatment under the NHS. While childlessness is distressing, it is not associated with long-term disability, morbidity or mortality. It is not a disease. Rather, it is about people unable to have something that they want. This is not what the NHS is there to remedy.^16^

Two main claims underlie Pemberton's comments; these will be explored in the subsections that follow. The first is that infertility should not be classed as a disease because it is only harmful to people *with certain desires*. The second is that infertility is a *social* problem but is mistakenly viewed as *medical* instead.

### Desire, not disease?

For individuals who do not want children, infertility is, at worst, a neutral characteristic and may even prove slightly advantageous, eliminating the need to worry about accidental pregnancy. Indeed, before the advent of effective contraception and safe abortion services, infertility would have been highly advantageous to any women who didn't want children (and still is in many parts of the world). Thus, *it may be argued that infertility is not a disease because its ability to cause harm depends upon people's preferences*.^17^

However, while infertility is only harmful when the sufferer is in possession of certain desires, the same is true for many diseases and disorders; infertility is by no means unique in this respect. Take colour-blindness, for example. Arguably, while there are everyday disadvantages associated with colour-blindness, the level of harm suffered will be relatively modest provided that the person lacks certain preferences. If, however, the person wanted to be an electrician, a painter or a pilot then (at least in some countries) her ambition would be thwarted with potentially serious consequences. Colour-blindness then, while always a disability or impairment, is only seriously harmful when certain desires are present, and in certain social contexts.^18^

A different kind of case in which desire and social context play an important role is where the infliction of injury confers sufficient benefit to make it worthwhile for the individual, all things considered. Perhaps the paradigm example of this is the ‘Blighty Wound’. A significant number of soldiers in World War I, faced with the prospect of death and disability, would inflict upon themselves a severe but not life-threatening injury in order to return home and avoid the front line. Horrifying cases have also been reported in which healthy young people have had limbs amputated in order to become more effective beggars, and trapped climbers have severed salvageable limbs in order to escape. In such cases, because of the person's desires and because of the social context, there may be no net harm to the individual, and there may even be benefits, all things considered. But we should nonetheless still say that the person has acquired a pathological condition, an injury or what Closer, Culver and Gert usefully term a ‘malady’.^19–21^

What follows from considering these cases? First, many pathological conditions are only harmful in the presence of certain desires. Hence, the fact that the major harms associated with infertility are dependent on the desire to have children does not mean that infertility cannot be a pathological condition. Second, many pathological conditions are only (directly) harmful in certain social contexts and may even be beneficial in others. So again, even if this is true of infertility, this does not mean that it cannot be a disease.

### Medical, not social?

A related suggestion is that infertility is in fact a *social problem* but is mistakenly viewed as *medical*.^22^ What might this mean?

One interpretation is that infertility is more like (say) bad housing, loneliness or poverty, than a disease. Perhaps this is what Pemberton has in mind when he speaks of how talk of ‘involuntary childlessness’, has given way to a medicalised discourse of ‘infertility’. The problem with this view though is that infertility usually *is* ‘medical’ in ways that bad housing and poverty are not. Its proximate cause is often biological, and the main option for alleviating it is medical treatment.^v^ This goes for infertility in general but applies especially forcefully to AUFI; it would be hard to sustain the claim that not having a uterus is a merely social phenomenon.

A second way of understanding the ‘social not medical’ claim is as the suggestion that infertility is more *difference* than *disorder.* One might, for example, compare infertility to being unusually short, or having a face or body-shape that does not conform to prevailing aesthetic norms. Characteristics like these, so the argument goes, are differences and not diseases. They are often disadvantageous to the person (and may sometimes even be ‘treated’—eg, through cosmetic surgery or human growth hormone). However, they are not diseases because the disadvantage associated with them is caused wholly or primarily by social discrimination. In cases like these, it may be argued that supporting medicalisation (by funding ‘treatments’) would be wrong because it would waste valuable resources and, perhaps more importantly, because to do so would be to collude with discrimination. For example, having darker skin in a racist (predominantly ‘white’) society is highly likely to be disadvantageous. Yet, while this is so, it would be both inefficient *and* a wrongful collusion with racism for the state to respond by providing a medical fix (such as skin whitening) for this social problem.

In the case of infertility, the parallel claim is that there is nothing *really wrong with* not being able to have children (just as there is nothing *really wrong with* being black, short, or ‘unattractive’). But, in our ‘pronatalist’ society, not being able to have children is disadvantageous because of discriminatory attitudes and practices. Some of these are general pro-parental ones applying to men and women alike such as the view that non-parents’ lives are inferior, with less meaning and purpose. Others more adversely affect women and reflect sexist attitudes, which encourage us to see women as essentially or naturally mothers, and as incomplete or defective if they remain childless.^22^ ^23^

So, is funding infertility treatment in some respects like providing cosmetic surgery so that people can fit in with society's aesthetic norms, and like skin whitening in response to racism? Our view is that, although this critique of the medicalisation of infertility is not wholly without foundation, it fails to provide a strong enough reason to deny funding for infertility treatments in general or UTx in particular. For, although the harmful effects of infertility are *made worse by* pronatalism and sexism, discrimination is not the sole cause of that harm, nor is it the case that there would be no harm if it weren't for the discrimination.

There are two reasons for this. As has already been noted, AUFI does involve the subnormal functioning (or absence) of a bodily part or process and does deprive women of the option to become pregnant, and this would be so even in a utopia without sexist and pronatalist attitudes. Regarding the desire to parent, we concede that this may be encouraged and influenced by such attitudes. It may be that more women want children, and that those who want children desire them more forcefully, because of prevailing ideologies. However, it is implausible to see such desires as *solely* caused by these ideologies; even in a society with no sexism and pronatalism people would still want children. *Fewer* perhaps would want them, maybe they would want them *less forcefully,* and crucially maybe those who couldn't have them would *feel less bad about it*. But it is implausible to suppose that a desire for parenthood is wholly down to pronatalism and sexism.

Infertility then is in a similar position to many (other) physical disabilities. Its proximate cause is what we might term impairment: the ‘malfunctioning’ of a bodily part or process leading to a lower than (statistically) normal level of ability. Sometimes this has direct negative effects (ones not dependent on social context) but other effects are caused or exacerbated by social context: disability discrimination, and in the case of infertility, pronatalism and sexism.^18^ ^24^ ^25^ Thus, however interpreted (and we have tried here to construct some sympathetic interpretations) the view that infertility is ‘social not medical’ is implausible; this is true *a fortiori* of AUFI, which has very clear biological underpinnings. Its negative effects may well be exacerbated by pronatalist and sexist ideologies but these are certainly not their sole cause.

Finally, we have allowed an assumption to be made in this section that should not be allowed to stand unchallenged: the suggestion that the state should *only* fund interventions that address disease. While this is not an issue on which we need to take a stand here, there may be sound policy reasons not to have this restriction. Imagine, for example, that it is entirely normal for people to have minor aches and pains: in other words, these are not the symptoms of disease but just part of the human condition (like feeling tired at the end of a busy day). If these aches and pains could be removed cheaply and beneficially through medication would there not be a case for state funding? Similarly, what about people who are anxious or shy but not sufficiently so for them to qualify as mentally ill? Again, if such things could be dealt with cheaply and beneficially through counselling or medication, would there not be a case for funding? We suggest therefore that there may be instances in which it is appropriate for the state to use its resources to address issues other than disease. And, if this is right, that would further weaken the argument which says that countries with socialised medical systems should not fund UTx because what it ‘treats’ is not a ‘proper’ disease. For even if it were not it could still merit ‘treatment’.

## The existence of sufficiently good alternatives

If it costs 10 times more to have a child via a uterus transplant than adoption or surrogacy, does that pregnancy have 10x the value? While we would never put a price on the mother-child bond, surely there is a higher opportunity cost with achieving a pregnancy with uterus transplantation than other ARTs?^26^

A third argument against funding UTx appeals to the requirement that even where cost-effectiveness thresholds are met by a particular treatment option, more expensive means of attaining some particular benefit should not be preferred to less expensive means of delivering the same (or a relevantly similar benefit) absent the provision of *compelling reasons* to the contrary.

As an illustration consider the following cases:

*Golden Fillings*: a dentist provides a patient with an expensive golden filling instead of a cheaper and similarly effective porcelain filling after the patient says that he particularly likes the colour and feel of gold in his mouth.
*Scalp Cooling*: a patient undergoing treatment for ovarian cancer is provided with scalp-cooling treatments in addition to the standard chemotherapy treatment provided for her form of cancer in order to reduce the likelihood of her experiencing chemotherapy-induced alopecia.

In both cases, decisions are made to provide more expensive treatments, despite the existence of cheaper alternatives, in order to provide additional benefits. Yet, although the additional benefits provided by *Scalp Cooling* are often considered to warrant additional funding—the NHS, for example, justifies the provision of scalp cooling because of the ‘significant threat to body image and self-esteem’^27^ that chemotherapy-induced alopecia poses to cancer sufferers—the supposed aesthetic benefits provided by *Golden Fillings* are not deemed to do so. The provision of a golden as opposed to a white tooth is, after all, neither necessary to keep one's ‘mouth, teeth and gums healthy and free of pain’^28^ nor held to be so central to individual well-being that its additional expense may be justified on similar grounds to scalp-cooling.

Thus, with such cases in mind, is UTx an appropriate candidate for funding in the light of the following facts?

1. *Women with AUFI are not without other options for parenthood*. All, provided they meet certain criteria, have the option to become social parents via adoption or traditional surrogacy arrangements, and many, whose infertility is not multifactorial, have the option to become genetic as well as social parents via gestational surrogacy arrangements.^vi^
2. *UTx is highly likely to prove the most expensive of such options*. UTx requires four surgeries^vii^, antirejection medication, at least one cycle of IVF and close monitoring of recipients for at least a year after transplant and throughout their pregnancies. Adoption however, has *no* medical costs, and while imposing some costs on social services may well *save* money by reducing the financial burden of support orphans impose upon the state. Indeed, although surrogacy is significantly more expensive than adoption,^viii^ such costs are liable to constitute a financial drop in the ocean compared with those associated with UTx.

So, is UTx, like *Scalp Cooling,* an example of an intervention which—although more expensive than the available alternatives—may be justified by reference to the additional benefits it provides? Or, should UTx, like *Golden Fillings*, be deemed to constitute a ‘frill’ or ‘optional extra’ that while desired by some is not of sufficient gravity to warrant the additional expense?

### The option of adoption

Of the three options considered here, adoption is the cheapest. Like UTx, adoption provides social parenthood. Yet it fails to provide benefits associated with UTx such as the opportunity to gestate and parent one's own genetic offspring. It also seldom offers the opportunity to parent a child from birth and may require a willingness to raise children with complex emotional needs and who are less likely to physically and/or psychologically resemble their parents.

Whether or not adoption should be seen as a ‘good enough’ alternative to UTx thus depends greatly on *which interests* state-funded infertility treatment is, and should be, designed to promote. If these are strictly limited to one: rearing children (social parenthood) then the benefits that adoption fails to offer, especially in cases where the child offered is an infant, may rightly be deemed to be ‘frills’ or ‘optional extras’. That is, while such benefits may be desired, these desires are not ones that the state has an obligation to satisfy. If, however, the accepted aims of state funding for infertility treatment are broader, including enabling individuals to found a family in the way *they desire*, the suggestion that adoption is a sufficiently good alternative is questionable.

Were the state forced to pick just one interest as providing the rationale for funding infertility treatment, a desire for social parenthood seems by far the strongest candidate. Social parenthood after all can provide life-long benefits to those who seek it and a transformative experience for which there are few (if any) substitutes.^32^ ^33^ Furthermore, few would suggest that funding should be provided to those who are infertile and wish to *procreate but not raise* a child. Yet, while this is so, one need not, in affirming the primacy of one aspect of parenting, necessarily deny the significance of others, or indeed, the significance of the journey to parenthood itself.

That the route taken in pursuit of a desire may be just as significant to an individual as its attainment can be illustrated by returning to *Motability,* a scheme providing adapted vehicles to persons with disabilities. Those who seek independence and interpersonal contact by way of the *Motability* scheme could achieve significant levels of both by the provision, not of a vehicle, but an internet connection. They may shop for groceries and clothing online, keep in contact with friends and family via social media and Skype and work or attend school/university remotely from home. Yet, despite this, it is far from clear that such online alternatives are ‘sufficiently good’. For the ability to try on clothing, pick groceries rather than having them picked by others, have coffee with a friend, rather than at home while on Skype, etc, are all, although not strictly necessary for independence or social interaction, goods liable greatly and positively to impact upon well-being.

As in *Motability,* where the social and experiential benefits afforded by cars, as opposed to internet connections, might justify the more expensive option, the same applies when comparing UTx and adoption. For, even if the primary aim of infertility treatment is the provision of a child, there seems little reason to assume that the social and experiential benefits many receive by gestating or being genetically related to their children may not go at least some way towards justifying the additional costs of UTx. These benefits can include experiencing pregnancy and birth, interacting with others *as* a pregnant woman, bonding with one's child prenatally, keeping parenthood and procreation ‘within the family’ and avoiding potential perceptions of rejection in the future should one's child seek out his ‘birth’ parents, carrying on a family line, and creating a physical manifestation of the love between two individuals.

As discussed in the preceding section, perhaps the desire for genetic and gestational parenthood is at least partly a result of cultural and social sexist and pro-natalist bias, which results in the unwarranted inflation of the significance of such desires.^34^ But even if this is so, the fact remains that until such time that this bias is eliminated (and, most likely after) the inability to have one's own genetic and gestational children will have significant and enduring negative effects on the welfare of many people.^35–38^ Indeed, regardless of one's feelings on this matter, it should be noted that adoption remains an alternative for virtually *all* infertile couples alike and, excepting perhaps cases of artificial insemination (which cost only £500–1000 per cycle^39^), is liable to prove more cost-effective than virtually *all* forms of ART. Thus, as in the case of the arguments dealt with in previous sections, if the availability of adoption constitutes a reason to withhold funding for UTx, it also, for reasons of consistency, provides a reason against funding most other fertility treatments, including IVF.

### Is surrogacy a sufficiently good alternative?

A more focused argument that might tell against funding UTx but not all other fertility treatments is the suggestion that surrogacy, unlike adoption, is a cheaper and sufficiently good alternative. For, although surrogacy fails to offer at least one of the benefits provided by UTx (gestation), the benefits it can offer—social parenthood from the moment of (or very soon after) birth and genetic relatedness—are far more closely matched to UTx than those offered by adoption. Given this, should we view surrogacy to be a sufficiently good alternative—one that women with AUFI can reasonably be expected to accept—in virtue of the extra costs associated with UTx?

The thought that surrogacy is ‘sufficiently good’ seems to depend on at least one of the following views.

1. Experiencing gestation is *relatively* unimportant compared with being a social parent from birth.
2. Experiencing gestation is *relatively* unimportant compared with genetic relatedness (ie, being genetically related to one's own children).
3. Experiencing gestation is no less important than genetic relatedness, or parenting from birth, but is nonetheless *insufficiently* important to justify the additional costs associated with UTx.

We will leave the last of these to one side, since it is not so much an objection in principle to funding UTx as an assertion that it is presently too expensive.

What about the first two positions though?

The first view, that experiencing pregnancy is relatively unimportant compared with the opportunity to parent a child from birth is initially plausible because of a point made earlier: that the primary purpose of ARTs is to deliver an opportunity *to parent* and we would (for example) find the idea of publicly funding ARTs for the purpose of enabling women *with no interest in parenthood* to experience pregnancy as bizarre and unjustified. That said, the importance of pregnancy must not be overlooked or understated for at least three reasons. First, as noted previously, the *experience of* pregnancy and childbirth is something to which many women attach tremendously high value and is often thought to facilitate ‘bonding’ with one's child. Second, some philosophers have argued that gestation is as important as, or more important than, genetic or social ties when it comes to establishing maternal status and maternal moral claims over the child.^40^ ^41^ Third, as we will discuss in a little more detail later, many jurisdictions, including the UK, have chosen to privilege gestation when deciding who the legal mother is at birth.^42^

So, although we concede that experiencing gestation is relatively unimportant when compared with social parenthood, gestation does still seem to be important in the ways outlined above. Thus, we might think of UTx as being more akin to *Scalp Cooling* than *Golden Fillings*. In *Scalp Cooling* nearly everyone would admit that retaining one's hair is *less* important than having one's life saved from cancer, yet few would view it as trivial. Similarly, we might say of gestation that it is *less* important than having a child to look after, but not unimportant. Hence, as in *Scalp Cooling*, paying more so that women can experience pregnancy may still be a legitimate candidate for public funding.

Similar considerations apply to the second view (the suggestion that gestation is *relatively* unimportant compared with genetic relatedness) and again it is not at all obvious that genetics should straightforwardly be privileged over gestation. For, although this view is prevalent in certain religious and cultural contexts^ix^, and would historically have been left unchallenged—due, most likely, to the role genetic relatedness played in legitimacy, inheritance and succession rules—there seems little reason to suggest that the contribution of the genetic material that provides the ‘form’ for a child is any more significant to parenthood than the contribution of the matter from which a child is constituted.^44–46^ A study undertaken in 1997 by Ravin *et al* who surveyed 106 infertile men and women of reproductive age in a fertility clinic in the US lends support to this suggestion. It uncovered that in relation to a preference for genetic or gestational parenthood where only one is available, while the majority of men preferred a genetic link to their offspring for obvious reasons, the opinion among female respondents was split 52/48% in favour of gestational parenthood.^47^

Finally, there are various practical reasons for thinking that surrogacy is not a sufficiently good alternative.

First, surrogacy inevitably involves an additional third party (the surrogate) in reproduction and, while many people's experiences of surrogacy are positive, this is not something that everyone will welcome.^48^

Second, surrogacy raises ethical issues that UTx, especially using deceased donors, does not. Many, for example, have raised concerns about consent and exploitation in both commercial and altruistic surrogacy arrangements^49^ ^50^ and others may hold that even in the absence of such concerns it is preferable for those who wish to procreate to undergo the risks associated with pregnancy and childbirth themselves rather than ‘outsourcing’ them to third parties.

Third, there is the difficult legal position of surrogacy in many countries. For although there are some states in which laws regarding both commercial and altruistic surrogacy are quite permissive, such as Russia,^51^ India,^52^ and Israel^53^ which permit both altruistic and commercial surrogacy arrangements, this is not the norm. Many states such as France,^54^ Germany^55^ and Switzerland^56^ ban the practice altogether, and others such as the UK,^57^ Canada^58^ and New Zealand^59^ permit only altruistic arrangements and hold surrogacy contracts to be unenforceable. This has led many to travel abroad from more restrictive to more permissive nations in order to secure a surrogate, generating legal problems such as stateless children.^60^ It has also, however, compounded concerns noted above regarding the exploitation of both surrogates and commissioning parents in those permissive countries that lack the resources or infrastructure to regulate the practice satisfactorily.^49^ ^50^

So, should we view surrogacy as a sufficiently good alternative, given the costs associated with UTx? As we have conceded, it may well be the case at present that the extra benefits delivered by UTx are insufficient to justify the considerable extra expense that it entails. However, if UTx became cheaper, the case for viewing it as more akin to *Scalp Cooling* than *Golden Fillings* could become sufficiently strong to justify funding. For, while the primary purpose of ARTs is to provide a child, the experience of pregnancy—although *less* important than social parenthood—is not *unimportant*, at least for those who desire it. In addition, we must keep in mind the far from ideal social and legal position of surrogacy in many countries. This makes surrogacy much less attractive to prospective parents than it could be and makes the demand that women with AUFI should ‘settle for’ surrogacy correspondingly less defensible.

## Conclusion

This paper has examined three arguments against publicly funding UTx.

The first, the Environmental Argument, claims that the state should not support assisted reproduction due to its obligation to prevent climate change and environmental pollution. This was rejected on grounds of fairness. The infertile should not pay a greater price than others when it comes to preventing population growth when fairer and likely more effective methods of discouraging reproduction are available, such as financial incentives.

The second, which holds that infertility is ‘social not medical’, was also rejected. While infertility (in common with most other disabilities) has social aspects, there are nonetheless good reasons for viewing it as a *bona fide* disorder. This is true *a fortiori* of AUFI, which has clear biological causes and effects.

The third claims that UTx should not be funded because of the existence of cheaper and ‘sufficiently good’ alternatives (adoption and surrogacy). This argument is partly about cost-effectiveness but also requires careful consideration of what counts as a ‘sufficiently good’ alternative. We suggested that, given adoption's inability to allow gestation or deliver a genetically related child from birth, there are reasons to think that adoption is not ‘sufficiently good’. Surrogacy however is more closely matched to UTx and, we concede, could prove ‘sufficiently good’ in some circumstances. In practice, however, surrogacy is dogged by various problems which mean that it is not, at least at the present time. Foremost among these is its legal position in many jurisdictions, which can cause uncertainty for, and make it difficult to employ and pay surrogates without going overseas. For reasons of this kind, the third argument also fails, at least in the present socio-legal context.

We conclude therefore that the case for ruling out state funding for UTx is weak. This does not mean that funding should actually be provided in the circumstances that obtain at present. For, just as with all other medical treatments, it must first be shown to be effective, safe and cost-effective. Also, surrogacy law reform could go a long way towards making surrogacy a ‘sufficiently good’ alternative and, if such reform occurred, the case for funding UTx would be significantly weakened.

## Footnotes

i The scope of this paper is thus limited to countries with socialised healthcare systems paid for through taxation and has little/nothing to say about countries where healthcare is all, or nearly all, privately funded. Similarly, general objections to performing UTx whether funded by the state or not—such as those relating to concerns about consent of both donors and recipients, the desires UTx caters to, or worries about harms and risks to donor, recipient and future offspring—are side-lined. Thus, we are assuming (at least for the sake of argument) that UTx would or could be clinically and ethically justified if funded privately.

ii The WHO currently estimates that 1.9% of women of ages 20–44 suffer from primary infertility and 10.5% of women suffer from secondary infertility. As only 1 in 500 women of childbearing age suffer from uterine factor infertility (UFI), it can be safely assumed that the demand for other forms of ART, including IVF will always be liable to far surpass demand for UTx even if 100% of women with UFI were desirous of both children and UTx. See ref. 10.

iii While CAS is a fictitious example, Motability is not. For more information see: http://www.motability.co.uk/

iv The Practice Committee of the American Society of Reproductive Medicine similarly tells us that: ‘Infertility is a disease’, defined by the failure to achieve a successful pregnancy after 12 months or more of appropriate, timed unprotected intercourse or therapeutic donor insemination. It goes on (following *Dorland's Illustrated Medical Dictionary*) to define ‘disease’ as: ‘Any deviation from or interruption of the normal structure or function of any part, organ, or system of the body as manifested by characteristic symptoms and signs; the etiology, pathology, and prognosis may be known or unknown’. See ref. 13.

v This is not to deny of course that lifestyle and social situation do not have a role to play here, as indeed they do in many aspects of health.

vi Here, traditional surrogacy refers to surrogacy arrangements in which the surrogate is genetically related to the child she gestates (ie, using her own ova and either donor sperm or the sperm of the intended father) and gestational surrogacy refers to arrangements in which the surrogate is not genetically related to the child she gestates (ie, where the ova of the intended mother or that of a donor is used).

vii Uterus retrieval, transplantation, caesarean section and ultimately removal from recipient.

viii Such costs generally involve the payment of expenses to a surrogate (estimated by Surrogacy UK to be around £8–15 000), the cost of her maternity care (averaging £2800 per woman in UK) and requires IVF where the gametes of both intended parents are used (around £5000 per cycle). See refs 29–31.
